# Application of Stable Isotope-Assisted Metabolomics for Cell Metabolism Studies

**DOI:** 10.3390/metabo4020142

**Published:** 2014-03-31

**Authors:** Le You, Baichen Zhang, Yinjie J. Tang

**Affiliations:** 1 Department of Energy, Environmental and Chemical Engineering, Washington University, St. Louis, MO 63130, USA; E-Mail: leyou@go.wustl.edu; 2 Plant Metabolomics Group, Institute of Plant Physiology and Ecology, Shanghai Institute for Biological Sciences, CAS, Shanghai 20032, China

**Keywords:** ^13^C-fingerprinting, flux, GC-MS, isotopologue, mass-to-charge, regulatory mechanisms

## Abstract

The applications of stable isotopes in metabolomics have facilitated the study of cell metabolisms. Stable isotope-assisted metabolomics requires: (1) properly designed tracer experiments; (2) stringent sampling and quenching protocols to minimize isotopic alternations; (3) efficient metabolite separations; (4) high resolution mass spectrometry to resolve overlapping peaks and background noises; and (5) data analysis methods and databases to decipher isotopic clusters over a broad *m*/*z* range (mass-to-charge ratio). This paper overviews mass spectrometry based techniques for precise determination of metabolites and their isotopologues. It also discusses applications of isotopic approaches to track substrate utilization, identify unknown metabolites and their chemical formulas, measure metabolite concentrations, determine putative metabolic pathways, and investigate microbial community populations and their carbon assimilation patterns. In addition, ^13^C-metabolite fingerprinting and metabolic models can be integrated to quantify carbon fluxes (enzyme reaction rates). The fluxome, in combination with other “omics” analyses, may give systems-level insights into regulatory mechanisms underlying gene functions. More importantly, ^13^C-tracer experiments significantly improve the potential of low-resolution gas chromatography-mass spectrometry (GC-MS) for broad-scope metabolism studies. We foresee the isotope-assisted metabolomics to be an indispensable tool in industrial biotechnology, environmental microbiology, and medical research.

## 1. Introduction

Metabolomics measures and interprets the time-related concentration and flux of endogenous metabolites in cells [1]. Metabolomics links our physiological knowledge from genome-type to phenotype by obtaining an instantaneous snapshot of final gene products (*i.e.*, metabolites). In metabolomics studies, extracellular metabolite profiles (metabolic footprinting or exometabolome) capture the features of overflow metabolism and offer a noninvasive approach to probe cells’ physiological alterations (nutrient uptake and metabolite secretion) in the appropriate media provoked by environmental or genetic perturbations [2]. On the other hand, the profiles of intracellular metabolites reflect gene functions in complex cell metabolism. Each type of organism may have unique intracellular metabolites (metabolite fingerprints) that elucidate specific cellular processes. By comparative analyses of metabolite profiling from mutant species, metabolomics can reveal enzyme activities, silent genes, and metabolic network topology [3].

Early metabolomics relies on NMR spectroscopy, which is widely used to study pathological factors and mechanisms (e.g., xenobiotic metabolism related to drug resistance in infectious diseases or cancer chemotherapy [4]). To reveal isotopologues or calculate isotopomers, multi-dimensional NMR is required and the analysis is quite time-consuming. Alternatively, mass spectrometry (MS) based metabolite profiling also plays an important role in investigating cell metabolism. Primary metabolites (e.g., amino acids, organic acids, and sugar phosphate) can be readily identified by MS and thus have become common targets in metabolism studies [5]. Meanwhile, new analytical instruments are developed with improved chromatographic separation efficiency, linear dynamic range of ion signals, and mass resolutions. Modern MS instrument is able to probe many low abundant metabolites, particularly secondary metabolites (e.g., hormones and natural products), and may generate comprehensive insight into genome-wide cell metabolisms.

The application of metabolomics still faces several limitations due to the large percentage of noise and artifacts of MS data, which render deciphering MS data a complicated process. Firstly, the loss and degradation of metabolites during sample quenching and separation make it difficult to precisely identify and quantify unstable metabolites. Secondly, unambiguous detection of putative isobaric or isomeric metabolites is still subject to technical difficulties. Third, direct metabolite profiling cannot reveal the actual enzyme activities since a metabolite pool is regulated by its carbon fluxes (enzyme reaction rates). Lastly, the high resolution MS is expensive and inaccessible to many research laboratories. In contrast, GC-MS offers an affordable platform, but has low metabolite detection power. It is desirable to improve the potential of GC-MS to obtain more physiological knowledge from limited metabolite datasets.

**Figure 1:**
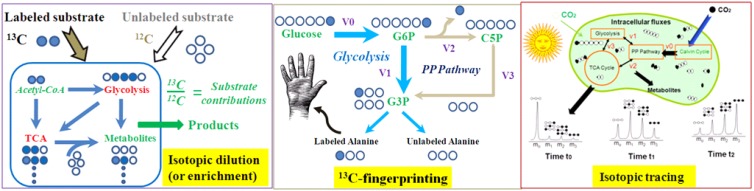
^13^C-labeling approaches for metabolism analysis.

To overcome such problems, isotope tracers are introduced to track the element fates in cell metabolites and delineate functional pathways [6]. Since the 1950’s, isotopes, particularly radiotracers, have been routinely applied to study enzyme reactions and are employed today to elucidate metabolic functions [7,8]. The wide application of stable isotopes (^13^C or ^15^N) in metabolomics was realized by Dr. Fiehn and his well-established plant metabolomics group [9]. Recently, the application has been extended to GC Time-of-Flight (GC-TOF) based metabolite determination [10] and elemental formula assignments [11,12]. Meanwhile, rooted in early isotopomer analysis of metabolites with GC-MS or NMR in 1980s, ^13^C-metabolic flux analysis (^13^C-MFA) is able to quantify *in vivo* enzyme functions [13,14,15]. Different tracer experiments (Table 1 and Figure 1), followed by isotopic analyses, allow the detection of metabolites and the quantification of intracellular metabolic fluxes in diverse organisms. More importantly, the ^13^C-fingerprinting of metabolites employs proper ^13^C-substrates to create special labeling patterns in key metabolites. By examining ^13^C-fingerprints in only a few abundant metabolites (such as amino acids), ^13^C-MFA models are able to deduce the functions of putative gene clusters and quantify global cell metabolism. For example, the biotechnology industries often use GC-MS based ^13^C-MFA to evaluate microbial hosts’ metabolism and nutrient utilizations [6].

Stringent sampling and quenching protocols, sufficient chromatographic separations and sensitive MS analyses to reduce background noises, and proper design of tracer experiments are required to minimize isotopic alternations in isotope-assisted metabolomics. In this paper, we discuss the use of isotopes (mainly ^13^C) in combination with metabolomics analysis to power the metabolic investigation of primary metabolites mainly based on GC-MS in microbial systems as examples. The sample treatment, platforms for separation, and the selection of MS that are needed to precisely determine metabolites and isotopologues are discussed in details. The practical applications of isotopes for broad-scope metabolism studies are also discussed. We foresee that stable isotope–assisted metabolomics, in combination with other “omics” tools, will not only provide a complete picture of cell physiology, but also decipher regulatory mechanisms underlying gene functions.

**Table 1:** Summary of isotopic labeling approaches [6].

Approaches	Description	Example
Isotopic dilution (or enrichment)	Grow cells with multiple carbon sources (some of them are labeled); then measure labeling of the metabolic products. This method is used for studying cell nutrient utilizations.	In a culture with ^13^C-glucose and yeast extracts, analysis of ^13^C-enrichment in proteinogenic amino acids reveals the contributons of yeast extract to biomass synthesis.
Isotopic tracing	Expose cell culture to a labeled compound (pulse); then measure change of labeling in downstream metabolites over time (chase). Pulse-chase tracing allows isotopic non-stationary MFA to quantify cell fluxomes [16].	The kinetics of isotopic incorporation from a nutrient into a downstream metabolite can detect and quantify functional pathways (e.g., kinetic flux profiling) [17]
^13^C-fingerprinting	Use specified labeled ^13^C-substrates to create steady state and position specific labeling patterns in metabolites, which delineate functional pathways. ^13^C-fingerprint allows ^13^C-MFA to quantify cell fluxomes.	If cell grows with 1st position labeled glucose, labeling patterns in serine and alanine can examine the Entner-Doudoroff pathway function.

## 2. Sample Preparation, Metabolite Separations, and MS Analysis

### 2.1. Metabolite Quenching

A proper sampling procedure is critical for metabolite analysis. Rapid quenching of biological samples is often required to catch the actual metabolic snapshot since cell metabolism (especially for microbes) responds to environmental changes within seconds, and thus sample preparation may introduce artifacts in isotopic data [18,19]. Metabolite quenching can be done with fast freezing, using liquid nitrogen or perchloric or nitric acid treatments [1]. To reduce the artifacts, cold methanol (−40 °C) is often used as a fast-quenching solution [18]. The methanol-quenched cells can be separated and lyophilized before metabolite extraction. The methanol quenching process may cause cell membrane damage [19] and drastic leakage of intracellular metabolites to the medium [20]. To minimize cell damage, cold glycerol-saline quenching solution is used [21]. Fast filtration, collecting cells by filtration under vacuum before quenching, is an alternative method to reduce metabolite loss [20,22]. To speed up this process and reduce metabolic perturbation, filter cultures were employed [23]. Cells grow on the filter paper placed on an agarose plate loaded with media and the quenching is achieved by quickly moving the filter paper from the plate to the extraction solution.

### 2.2. Metabolite Extraction

Small metabolites have to be isolated from biological samples or tissues before analyses. Metabolite extraction methods are species, agent, and technique dependent. Certain metabolite classes may need additional workups for enrichment or removal of interferences. This is particularly important for some low abundant and critical central metabolites. Interference background sometimes impairs isotopologue calculations. Even for metabolites belonging to the same class, the efficiency of different extraction methods may vary significantly. Efforts have been made to identify the optimal metabolite extraction protocols for various biological systems [19,24,25,26]. The five most widely used extraction methods—hot water, boiling ethanol, chloroform-methanol, freezing-thawing in methanol, and acidic acetonitrile-methanol—were tested using *S. cerevisiae* cells. In general, boiling ethanol and chloroform-methanol methods have the best performance in terms of completeness of extraction, prevention of metabolite conversion, and metabolite stability [25]. Moreover, twelve different extraction methods for mammalian cells were evaluated by extracting metabolomes of Chinese hamster ovary cell line [27]. The cold extraction method with 50% aqueous acetonitrile was shown to be an optimal choice for mammalian cells.

### 2.3. Metabolite Derivatization

Chemical derivatization transforms metabolites into gaseous products to improve their detection by chromatography and MS. For GC-MS measurements, chemical derivatization increases volatility or decreases polarity of compounds that otherwise are not readily separated by GC prior to MS detection. Given the increased popularity of LC-MS systems, chemical derivatization may (1) improve the stability of unstable functional groups; (2) increase ionization efficiency and enhance detector responses of target compounds; (3) improve chromatographic separation; (4) aid in selective identification or enrichment of target analytes [28]; (5) generate characteristic MS-MS fragments of target metabolite classes to improve isotopomer determinations [29]. The chemical structure and properties of the metabolites will directly influence the derivatizing reagent choice. The most widely used derivatization reactions for GC-MS today are acylation, alkylation, and silylation [30]. The choice of derivatizing reagent is highly dependent on the structure of the functional group requiring derivatization and the selected MS technique. In ^13^C-MFA studies, two classes of silylation reagents are often used for metabolite derivatization: those generating trimethylsilyl derivatives (TMS) and those introducing *tert*-butyldimethylsilyl derivatives (TBDMS).

### 2.4. Metabolite Separation Platforms

The choice of the separation platforms is based on properties of metabolites of interest. GC is one of the most common technology platforms in metabolomics analysis. GC can be coupled with electron ionization (EI) sources that are less susceptible to ionization suppression. It provides accurate and low-cost measurement of sugars, amino acids, organic acids, and lipids [31]. Nonvolatile or thermally unstable metabolites (e.g., nucleotides and acyl-CoAs) cannot be directly separated by GC. Liquid chromatography (LC) is needed. Though LC peak qualities are lower than those of GC, LC separation covers a larger scope of metabolites including high molecular weight and thermally labile compounds. New technologies employing higher pressure pumps and smaller particle sizes in the LC columns lead to Ultra Performance (or Pressure) Liquid Chromatography (UPLC) with improved resolution and sensitivity, which are used to separate low abundant and unstable free metabolites. However, the conflicts among high peak capacity, LC resolution, and speed of MS detection have yet to be solved completely.

**Figure 2:**
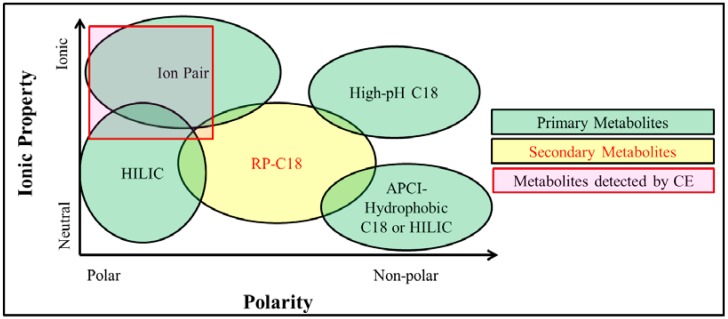
Coverage diagram of LC and CE metabolomics separation platforms.

Selection of LC separation columns depends on the polarity and ionic properties of metabolites (Figure 2). HILIC (hydrophilic interaction chromatography) probes a portion of metabolomes including neutral saccharides and ionic glycan [32]. Secondary metabolites are usually moderately hydrophobic, and Reverse Phase Chromatography (e.g., RP-C18 LC column) performs best [33]. Ion Pair High Performance LC (HPLC) [34] can resolve majority of nucleotides, coenzyme A esters, sugar nucleotides, sugar bisphosphonates, and some key intermediates in secondary metabolism [35]. High pH based C18 LC column can analyze ionic and hydrophobic metabolites from both lipid metabolism [36] and secondary metabolisms (e.g., alkaloids) [37]. For separation of less ionic and hydrophobic metabolites [38,39], Reverse Phase Chromatography (RP-C18 column) with stronger solvent systems other than methanol and acetonitrile should be used [40,41,42,43]; HILIC or normal hydrophobic LC column may also be applicable [44]. For example, a combination of Reverse Phase Chromatography and HILIC separation improves lipidome analyses [45,46]. It should be noted that electrospray ionization MS is unable to detect hydrophobic metabolites with none or few ionizable functional groups. Atmospheric pressure chemical ionization (APCI) MS should be coupled to LC for the measurement of such metabolites (Figure 2) [47].

Capillary Electrophoresis (CE), a fast separation technique, separates charged species with different electrophoretic mobilities in an electric field through a conductive medium. Its separation efficiency depends on capillary length and buffer solutions. CE coupled with electrospray ionization (ESI)-MS is suitable for the analysis of highly polar and ionic compounds [48]. It should be noted that the flow rate and sample loading capacity of CE are low, and thus sensitive ESI-MS devices are essential for wide applications of CE-ESI-MS. Ion chromatography coupled with MS can be an alternative to CE-MS, and an application for measuring 16 hexose-phosphates (hexose-Ps) and nucleotide-sugars has been reported [49]. Selection of LC and CE for metabolite separation depends on system availability, affordability, and analytical targets. For example, CE-MS in combination with LC-MS has been employed to obtain profiling of ^13^C-labeling of amino acids and central metabolites in a lysine-producing *Escherichia coli* to reveal culture phase-dependent metabolic shifts [50].

## 3. Isotopologue and Isotopomer Analysis

### 3.1. MS Resolutions

Isotopomers are isomers with the same number of each isotopic atom but differ in position [51], whereas an isotopologue is a molecular entity that differs only in “isotopic composition” [51]. Reliable measurement of metabolites and their labeling patterns depends on MS resolution (*i.e.*, the capability to distinguish two MS peaks of slightly different *m*/*z* ratios). For low to medium resolution MS, metabolome analysis requires chromatographic separation. However, ultrahigh-resolution MS may perform direct infusion based methods (*i.e.*, detection without metabolite separations) [52], leading to high throughput analyses of isotopologue profiles of amino acids [53] and nucleotides [54].

To understand the MS resolution for isotopologue profiling, we need to mention the trivial mass differences in isotopologue profiles for each metabolites. For ion signals of a metabolite with a general formula C_x_H_y_O_z_N_r_P_s_S_t_ [55], the formation of isotopic peaks is determined by the number of each element with natural isotopes and isotopic MS distribution [56]. Since ^13^C comes with almost 1.1% natural abundance, it dominates the isotopic profiles among the contributions of isotopic mass from other stable isotopes, including deuterium (D), ^18^O and ^15^N (note: phosphorus comes with no stable isotope). For example, in a ^13^C experiment for a compound with chemical formula C_8_H_10_NO_6_P, the major isotopologues will include M + 0 to M + 8 from zero to eight ^13^C carbons. Other contributions will include M + 1 from ^15^N, M + 2 from ^18^O, M + 4 from 2 ^18^O, and M + 1 from D. All other contributions will be minor due to their lower natural abundance. Table 2 shows the exact mass differences in these isotopes. Using the combinatorial differences, we can deduce the required minimum resolution to resolve isotopologues of each metabolite.

**Table 2:** Major contribution to the isotopic mass difference.

Mass difference (in Dalton)	^13^C–^12^C	^15^N–^14^N	^18^O–^16^O	D–H	^34^S–^32^S
	1.003355	0.997035	2.004246	1.006277	1.995796
Mass shift					
M1	1.003355	0.997035	-	1.006277	-
M2	2.006710	1.994070	2.004246	2.012554	1.995796
M3	3.010065	2.991105	-	3.018831	-
M4	4.013419	3.988140	4.008492	4.025108	7.983184

Note: each column indicates the exact mass shifts with the increase of the numbers of isotopes.

According to previous report, mass resolution calculation is based on the closest distinguishable separation between two MS peaks of equal height and width, which is a function of *m*/*z*, mass peak width, and the relative mass abundant ratios of two mixed isotopologue peaks [57]. A convenient equation below can estimate the minimum resolutions for MS analysis. For a rigorous measurement, the *Constant* can be assumed as ten [57].


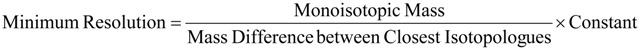
(1)

**Figure 3:**
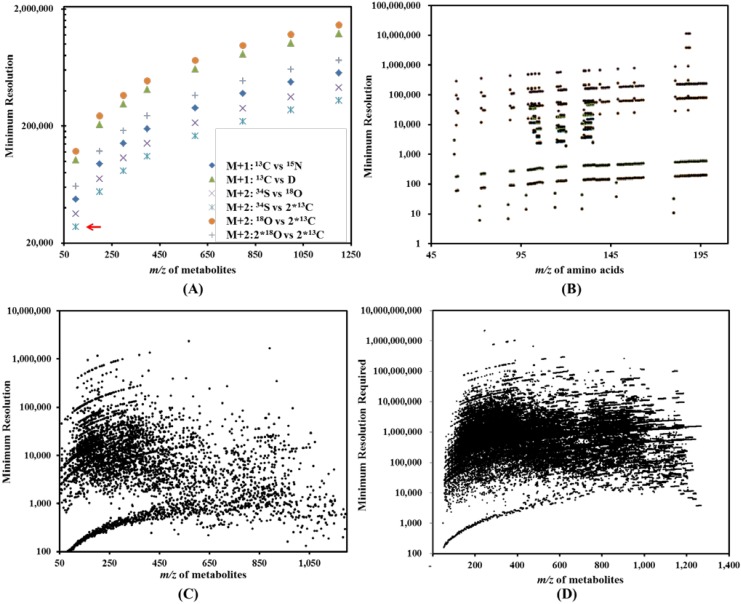
Resolutions required for metabolomics analysis [57]. Panel (**A**) shows the resolutions required to distinguish major isotopologues for metabolites of different *m*/*z*. Panel (**B**) shows the resolution required to resolve major isotopologues in amino acid mixture. Panel (**C**) shows resolution required to resolving isobaric masses in hypothetical metabolite mixture without considering isotopologues or isotopic peaks. Panel (**D**) shows resolution required to resolve common isotopologues of hypothetical metabolite mixture.

If the *Constant* in Equation (1) is set as three (assuming a less rigorous MS measurement), we plot the minimum resolutions required for isotopologue analysis of hypothetical metabolites (Figure 3). As an example, the dot beside the red arrow in Figure 3A represents metabolite fragments with *m*/*z* of 100, which may contain one ^34^S or two molecules of ^13^C. To differentiate between the M + 2 results from either one ^34^S or from the two ^13^C, a minimal resolution can be calculated as 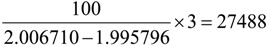 (see Table 2). Thereby, we have one dot at the x-y coordinates (100, 27,488) in Figure 3A. In a similar approach, the cloud of dots in Figure 3A represents the resolutions required to distinguish major isotopologues for metabolites of different *m*/*z*. Figure 3B reports the minimum resolutions required to analyze isotopologues from a mixture of amino acids (121 data points). Figure 3C (4,598 data points) represents the minimum resolutions required to resolve two isobaric metabolites (metabolites with the same molecular weight) in a mixture of hypothetical metabolites without considering effects due to isotopologues (or isotopic peaks). Similar to Figure 3B, Figure 3D reports the minimum resolution required to resolve close isotopologues (160,253 data points) in a mixture of hypothetical metabolites.

For metabolites with mass below 300 *m*/*z*, Orbitrap, QExactive Plus, and FT-ICR platform can reach the capacity to resolve the isotopologues of individual metabolites provided no overlapping signals from other metabolites or background ions. However, when the *m*/*z* increases to 800–1,200 *m*/*z* (in the range of lipid metabolites), a resolution over 400,000 is required, a feat still hard to realize with most available high resolution MS. The simulated requirement of resolution for amino acid mixture is provided in Figure 3B. Despite the past successes of resolving or partially resolving the isotopologues of a mixture of common amino acids, such as those from protein lysates, the requirement of resolution is still well beyond the reach of the majority of TOF (Time of flight) instruments, which has a maximum resolution between 20,000 and 100,000. Therefore, though the complete resolving of isotopologues in metabolite mixtures without separation is ideal, the required resolution renders it impossible to fulfill this goal with any MS that has been proposed to date (Figure 3C,D).

### 3.2. MS Platform Selection

MS platforms for metabolomics studies should be chosen based on research purposes and affordability. Medium to low resolution MS, such as traditional GC-MS, Triple-Quadruple instruments and some TOF instruments, Q-Exactive or Orbitrap may analyze isotopologue profiles in the aid of chromatographic separation. The GC-MS is particularly good for studies on central metabolism by measuring amino and organic acids using automated standard library searches. A system evaluation of GC-MS, LC-TOFMS and LC-MS-MS has indicates that low-cost GC-MS with an electron ionization source still provides satisfactory isotopologue analysis of many metabolites and the GC-MS datasets can be directly implemented in fluxomics software [58].

Metabolimcs sciences are continously growing and new MS tools are rapidly developed for metabolite identification and quantification [59]. Ultra-high resolution instruments, such as FT-ICR-MS, can perform direct infusion MS (DI-MS) to probe organic acids and carbohydrates from biological samples [52]. However, due to isobaric interference and a large number of isomers, many ions shown in DI-MS cannot be differentiated. For example, over 57% of metabolites from a summary of 7,876 compounds (MW between 50 and 1,200) recorded in MetaCyc [60] and PlantCyc databases have isomeric counterparts [61]. In an extreme case, there are 40 isomers recorded for the commonly occurring hexoses with a formula C_6_H_12_O_6_. No report to date has addressed the challenges of resolving all of isotopomers in one assay. As a consequence, chromatographic separation of metabolite mixture will still dominate future ^13^C- metabolomics.

MS-MS-based platform is a powerful metabolomics tool. High resolution full scan spectra can perform putative identifications of metabolites, and the subsequent MS (MS^2^) is used to confirm molecular structure. To obtain the high-quality MS^2^ data and remove unreliable fragments due to instrument noise and high complex sample compositions, a two-part approach for performing metabolomic identifications has been proposed, and a data analysis package (decoMS^2^) implementing the algorithms in the workflow can obtain deconvolved MS^2^ spectra for many biological compounds [62]. Alternatively, the high-resolution UPLC-TOFMS^E^ (E represents collision energy) in combination with mass defect filtering can identify both expected and unexpected metabolites from a single LC/MS acquisition [63].

## 4. Data Analysis

Processing ^13^C-metabolomics data to obtain the isotope enrichment or the position of the isotope in the molecules depends on MS systems and the tracer experiment design. There is no universal software tool available for this purpose. When a single isotope label is used, it is not necessary to require resolving various isotopologues from different natural isotopes. Instead, it is common practice to use low resolution MS instruments. In this case, isotopologues originating from various abundant natural isotopes (such as ^13^C, ^15^N, ^18^O *et al.*) are convoluted in isotopic clusters. Enriched isotopic abundance can be deducted after removing the natural isotopic abundance by mathematical algorithms [64]. For isotopomer analysis, MS fragments are necessary. Even for isotopomers from a single type of isotopes, such as carbon, the possible number of isotopomers (2^n^: n represents number of elements in a chemical formula) can be quite large. Therefore, the complete calculation of isotopomers is possible only when n is small and enough MS fragments are available with fair abundance.

Usually MS-MS spectra need to be extracted for subsequent correction of natural isotopic abundance [65]. For LC-MS-MS with low resolution MS instruments such as Qtrap, an exhaustive MRM (multiple reaction monitoring) list can compute possible isotopomers if enough MS-MS fragments are detectable [66]. Recent versions of commercial triple quadruple MS may allow over 3,000–4,000 MRMs programmed and will be beneficial to this type of experiment. However, MS analysis may not distinguish isotopologues if multiple isotopes are present in the sample [67]. Moreover, the convolution of isotopologues, isobaric overlapping, and background interference in MS measurement may dramatically decrease its sensitivity to detect small percentage of isotopic enrichment. ESI noises are notoriously difficult to control. For ultra-high resolution MS, preemptively correcting natural isotopic abundance can avoid convolution of isotopologues [68]. However, it is problematic when the instrument can only partially resolve the isotopologues. Partially separated isotopologues may complicate the peak extraction algorithms, mass accuracy, and isotopic correction. The situation is more complex if structurally uncharacterized metabolites are present in the samples, which are common in metabolomics experiments. Academic agreement has yet to be reached on how labeled metabolomics data from poorly characterized metabolites should be presented.

Many MS vendors provide data analysis tools for metabolomics. There are several options for analyzing the large datasets from metabolomics experiments [61,69]. (1) Vendor supplied software, such as ChromaTOF (LECO Pegasus GC-TOF, Saint Joseph, USA) software, MassHunter (Agilent TOF systems, Santa Clara, USA), Orbitrap (Thermo Fisher Scientific, Waltham, USA), and Sieve (QExactive, City, Country); (2) Third-party software is also available. For example, Non-linear Dynamics released Progenesis CoMet for analyzing LC-MS data; (3) Database and profiling software released from academic laboratories, such as XCMS [70], mzMine [71,72], and the METLIN Metabolomics Database [73]. However, commercial software does not meet the requirements to analyze isotopically enriched metabolomics data. Unresolved isotopologues from high resolution MS may block automated data processing. Automated MS/MS fragment interpretation is even more challenging. Reliable peak extraction is convoluted by partially resolved isotopologues and overlapped metabolites. Background MS noises add additional complexity. Current chromatography cannot completely resolve overlapped metabolites. Therefore, reliable data analysis methodologies for isotopically labeled metabolomics datasets are still under further development and require additional efforts from individual research laboratories as well as collaborations from commercial vendors, third party software suppliers, and an open source community. A uniformed algorithm collection or workflow with options flexible for instrument systems is advantageous. More importantly, a blueprint for validating various data analysis algorithms will help the researchers to pick the right software to analyze the labeled metabolomics dataset and avoid pitfalls for data analysis.

## 5. Application of Isotope-Assisted Metabolomics

### 5.1. Isotope Analysis for Tracking Nutrient Utilizations

A rich medium contains bottleneck nutrients to promote cell growth and product synthesis. When a ^13^C-labeled substrate is supplied in rich mediums, the metabolites synthesized *de novo* from the substrate will be labeled, while metabolites derived from the undefined nutrients in the medium are not (Figure 1). Therefore, ^13^C-labeling in biogenic and exogenous metabolites can identify the essential nutrients that are not effectively synthesized from primary substrates [74]. This method has particular value in medical research for disease diagnoses and pathological analysis. This method has also been used to identify the bottleneck nutrients for alkaline protease synthesis in an industrial microbial host [75]. In another example, the favorable nutrients for a slow growing *Dehalococcoides ethenogenes* (doubling times ~2 days) were investigated. ^13^C-dilution of proteinogenic amino acids using uniformly labeled acetate in a rich medium identified that only four amino acids are useful for promoting *Dehalococcoides* growth [76]. Moreover, ^13^C-labeling can also reveal the co-metabolism of multiple carbon substrates [77] as well as photomixotrophic biosynthesis processes [78].

### 5.2. Isotope-Assisted Metabolite Identifications

Isobars (molecules with same mass but different chemical compositions) may have trivial mass differences. However, their elemental composition may differ significantly. In terms of biogenesis, isobars originate from completely different metabolic pathways. By dosing specific labeled precursors, isobars can be resolved due to differences in isotopic enrichment. It is particularly self-evident when one isobar does not undergo isotopic incorporation and another does. Thereby, isotope labeling can help the unambiguous identification of metabolites. Differentiation of isobaric compounds or isomers (molecules having the same chemical composition but different chemical structures) requires sophisticated MS techniques to manipulate electric fields and design chemical ionization reactions to provide analytical selectivity [79,80]. Since labeled isotopes are distinguishable from abundant natural isotopes by *m*/*z* ratios, ^13^C and ^15^N labeling of biological samples often assist metabolite analysis [81]. By comparing exact MS data from metabolites resulting from different combinations of C and N tracers, high-confident metabolite identifications of isobaric compounds with different elemental compositions can be easily achieved [79,80,81,82,83,84,85,86]. For example, using a combination of ^13^C and ^15^N, untargeted metabolite profiling has identified cyanobacterial metabolites that are not annotated by its genome databases [83]. Besides, ^13^C and ^15^N labeling has also been used to differentiate or identify the metabolites from contaminants (*i.e.*, compounds of non-biogenic origin). For example, ^13^C-labeling and FT-ICR MS analysis have detected phytoplankton metabolic footprints in the seawater and improve our understanding of the role of exuded compounds from phytoplankton in an ecological system [84].

### 5.3. Isotope-Assisted Metabolite Quantification

Enzymatic and chromatographic methods cannot precisely measure concentrations of low abundant intracellular metabolites. To overcome this problem, the use of isotopically labeled metabolites as internal standards can assist MS analysis to determine metabolite concentrations under the noise of sample degradation and instrumental variations [87]. Since standards of ^13^C-metabolites are expensive, the isotope dilution technique applies a modified inverse labeling approach [23]. With this approach, cells are grown in fully labeled substrates to yield exclusively labeled intracellular metabolites. Then, unlabeled internal standards in known concentrations can be mixed with the labeled cell samples during metabolite extraction. MS analysis of isotopologue data can obtain the ratio of endogenous metabolites to internal standards from the co-extracted sample. Absolute metabolite concentrations on the basis of standard concentrations can then be calculated [23]. This approach has been extended to determine metabolite turnover rates [17]: after cell cultures undergo a step change from unlabeled to ^13^C-labeled nutrient, the rate of ^13^C enrichments in a metabolite multiplied by its pool size gives the metabolite turnover rate (*i.e*., flux). Such kinetic flux profiling can probe pathway responses to diverse growth conditions [88] and discover the functions of enzymes in pathogens [89].

### 5.4. Isotope-Assisted Pathway Investigations

^13^C-tracer experiments are widely used to confirm or discover functional pathways [6]. For example, a threonine-independent route for isoleucine synthesis was found in a cyanobacterium by using 2-^13^C glycerol to fingerprint its metabolites [66]. Based on labeling features for threonine, isoleucine, and leucine, a citramalate pathway was discovered as an alternate isoleucine synthesis pathway in this strain. Moreover, the ethylmalonyl-CoA pathway in *Methylobacterium extorquens* was discovered by monitoring label-incorporation from 1-^13^C acetate into the pathway intermediates over time [90]. In the metabolic engineering field, ^13^C-labeling can determine the contribution of a certain pathway in the synthesis of a product and confirm the engineered pathway functions. For example, a recent study infers that ^13^C-labeling may examine a non-oxidative glycolytic cycle in *E. coli* that breaks down glucose into acetyl-CoA without carbon loss [91]. Besides pathway investigation, ^13^C tracer experiments can determine the physiological role of a functional enzyme by detecting the ^13^C profile in a corresponding mutant [77]. The role of malic enzyme for pyruvate synthesis in *S. cerevisiae* was determined by tracking the labeling patterns of pyruvate in the isogenic strain when the malic enzyme was deleted [77]. In addition, the understanding of metabolic pathways and their regulations is important in biology and biomedical fields [92,93,94,95,96]. For example, cancer cells have different metabolic characteristics, such as fast cellular proliferation, increased local invasion, and metastases from their normal counterparts [97,98]. ^13^C isotopic labeling can identify the metabolic pathway changes in the central metabolism that lead to the altered metabolic phenotypes in cancer cells [99,100,101,102]. Such analysis can reveal promising targets for future cancer therapies [103].

Stable isotope tracing has also been used for elucidation of network-wide metabolic pathways. For example, to analyze the glutamine metabolism in A549 human lung carcinoma cells, in which a compound library was first obtained using unlabeled cultures [85]. Then the labeled tracer (U-^13^C glutamine or amine-labeled α-^15^N glutamine) was supplied to cell culture with unlabeled glucose to fingerprint downstream metabolites. The mass isotopomer distributions of broad-scope metabolites provide critical confirmation of carbon fluxes percolated through the metabolic network. Such untargeted tracer fate detection offers great potential to study biochemical reactions that are currently unknown. The high-resolution MS and the advances in genome databases have greatly improved the extent of untargeted analyses of network-wide pathways [86].

### 5.5. ^13^C-assisted Metabolic Flux Analysis

Metabolic responses to genetic or environmental modifications can be monitored based on the steady state ^13^C-MFA [104,105,106], which often quantify fluxes based on ^13^C-fingerprints in proteinogenic amino acids [105]. ^13^C-MFA can also reveal algal mixotrophic metabolism using both CO_2_ and organic carbon substrates [107]. However, steady state ^13^C-MFA is not able to reveal the photoautotrophic metabolism or the dynamic metabolic responses. The advances in LC-MS techniques facilitate the development of isotopic non-stationary MFA (INST MFA) by capturing isotopomer dynamics of fast turnover intracellular metabolites [16,108]. Nowadays, INST MFA can analyze not only microbes, but also plant and mammalian cells [109]. In the biotechnology industry, non-stationary ^13^C-metabolic flux ratio analyses via isotopic tracing have obtained the snapshots of the metabolic fluxes through several key nodes in *B. subtilis* [108]. Time-dependent fluxomics has also been proposed to investigate *B. subtilis* fermentation processes [110].

^13^C-MFA models for mammalian and plant cells are much more complex than bacterial models in several ways. Firstly, the subcellular compartments of mammalian cells are highly active in carbon and energy transportation. The measurement of compartmented metabolites is difficult due to their small pool size and high leakage [111]. Secondly, during eukaryotic cell cultivation, it is difficult to ensure metabolic steady-state conditions for metabolic flux analysis [112]. Thirdly, complex media, essential for mammalian cell growth, includes undefined nutrition that encumbers metabolic flux calculations. Fourthly, the analysis of plant autotrophic metabolism requires INST MFA [16], and involves costly LC-MS analysis of low abundant and unstable free metabolites. Lastly, metabolite channeling and compartmentation may occur in eukaryote cells, which pass intermediates from enzyme to enzyme without reaching cellular medium equilibration. This alters the isotopomer composition of downstream metabolites [113] and creates high noises for flux estimation. Nevertheless, extensive efforts have been made to determine the metabolic functions in plant and mammalian cells via isotopic labeling [114,115,116], in which Chinese hamster ovary (CHO) cells are most well studied using flux analysis approaches [117,118,119].

### 5.6. ^13^C-asssited Metabolic Analysis of Microbial Communities

Species in microbial communities has complex nutrient interactions [120,121]. In ecology, stable isotope is widely used to determine microbial community populations and carbon assimilation patterns. For example, after feeding ^13^C-toluene to a microbial consortium, toluene-degrading microorganisms were identified by analyzing ^13^C-fatty acids (via GC-combustion-isotope ratio MS) and sequencing ^13^C-labeled 16S rRNA [122]. Moreover, ^13^C or ^14^C-approaches have been used to examine the metabolic interactions between microbial pathogens and their hosts [123]. For example, ^13^C-labeling can study the pathways used by intracellular pathogens or their mutants to determine nutrient utilization during infection [124,125]. Currently, actual metabolisms among community species are still difficult to investigate. It is difficult to obtain metabolites from a given species within a mixed culture because it requires complete separation of this species by repeated centrifugations or fluorescence-assisted cell sorting. To overcome this problem, a proof-of-concept for metabolite isolation from a community system has been proposed [126]: a “reporter protein” that has been synthesized in only one species of the consortium can be separated via chromatographic protein purification. Then ^13^C patterns of proteinogenic amino acids from the reporter protein can be used in isotopic fingerprinting.

## 6. Future Directions and Conclusions

**Figure 4:**
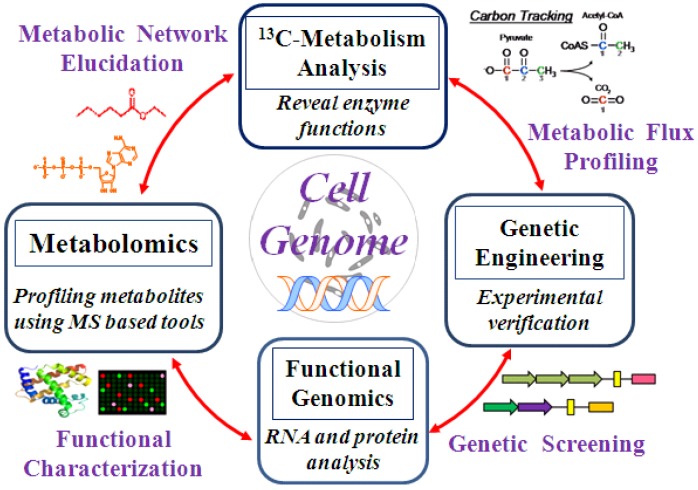
Metabolic knowledge mining by constructing an iterative method to interpret multiple omics data sets.

Isotope labeling has played an important role in studies regarding functional characterization of the cell genome and provided insights into cell physiologies. However, there are still many areas of cell metabolisms and their multiple level regulations are unknown. Systems profiling of messenger RNAs, proteins, and metabolites have been shown to offer a comprehensive insights into the intracellular activities and their regulatory network [127]. Recently, transcriptomics, proteomics, and metabolomics are extensively used to obtain a systematic insight into the multiple cellular processes and genetic regulations [128,129,130,131], where the metabolomics is the key to link multiple omics data for functional genomics studies [132]. Due to the putative post-transcription regulation, the correlation among genomics, transcriptomics, and metabolomics data is still not straightforward. For some genes, increases in mRNA levels may not lead to the increase in protein levels; for other genes, the mRNA levels are the same value but the protein levels vary significantly [133]. Moreover, the existence of isoenzyme and poor enzyme specificity contribute to high metabolome diversity (“too few genes, too many metabolites” [134]), which hinders the linkage between genomics and metabolomics. Therefore, the integration and application of multiple omics data sets is still in its preliminary phases and requires iterative “omics” analysis and experimental verification (Figure 4).

Isotope-assisted metabolomics is undergoing four technique developments. First, rapid MS detection without a separation stage can speed up screening of large biological samples, but it is only applicable for certain classes of metabolites or simplified metabolite mixtures. This is seen in nanostructure-initiator mass spectrometry (NIMS)-based approaches for screening targeted metabolic compounds and provides an important tool in characterization of enzymes from environmental microbes [135,136]. Second, MS analysis must include extensive efforts to generate information-rich MS-MS fragments with collision-induced dissociations in order to completely resolve isotopomer distributions. Otherwise, NMR techniques have to be applied for assisting MS for molecule structure analysis [137,138]. To overcome the challenges for separation and identification of isobars, UPLC and ultra-high resolutions MS are proposed to separate and detect very small mass differences among isomers [139]. Third, multiple isotopic labels (^2^H, ^13^C, and ^18^O tracers) can be used simultaneously to obtain maximum metabolic information in complicated networks. Fourth, by knowing only a few isotopologues in key metabolites, isotopomer tracing models (*i.e*., algorithms used in ^13^C-MFA) may deduce the complete isotopomer compositions resulting from labeled substrates [140]. Above-mentioned developments will ultimately expand the horizon of isotope-assisted metabolomics research, which measure and interpret the time-related metabolite concentrations and enzyme activities in an unprecedented manner.
